# Generation of High Current Densities in Geobacter sulfurreducens Lacking the Putative Gene for the PilB Pilus Assembly Motor

**DOI:** 10.1128/Spectrum.00877-21

**Published:** 2021-09-29

**Authors:** Toshiyuki Ueki, David J. F. Walker, Kelly P. Nevin, Joy E. Ward, Trevor L. Woodard, Stephen S. Nonnenmann, Derek R. Lovley

**Affiliations:** a Department of Microbiology, University of Massachusetts-Amherst, Amherst, Massachusetts, USA; b Institute for Applied Life Sciences, University of Massachusetts-Amherst, Amherst, Massachusetts, USA; c Department of Mechanical and Industrial Engineering, University of Massachusetts-Amherst, Amherst, Massachusetts, USA; University of Minnesota

**Keywords:** electromicrobiology, *Geobacter*, protein nanowires, extracellular electron transfer, conductive pili

## Abstract

Geobacter sulfurreducens is commonly employed as a model for the study of extracellular electron transport mechanisms in the *Geobacter* species. Deletion of *pilB*, which is known to encode the pilus assembly motor protein for type IV pili in other bacteria, has been proposed as an effective strategy for evaluating the role of electrically conductive pili (e-pili) in G. sulfurreducens extracellular electron transfer. In those studies, the inhibition of e-pili expression associated with *pilB* deletion was not demonstrated directly but was inferred from the observation that *pilB* deletion mutants produced lower current densities than wild-type cells. Here, we report that deleting *pilB* did not diminish current production. Conducting probe atomic force microscopy revealed filaments with the same diameter and similar current-voltage response as e-pili harvested from wild-type G. sulfurreducens or when e-pili are expressed heterologously from the G. sulfurreducens pilin gene in Escherichia coli. Immunogold labeling demonstrated that a G. sulfurreducens strain expressing a pilin monomer with a His tag continued to express His tag-labeled filaments when *pilB* was deleted. These results suggest that a reinterpretation of the results of previous studies on G. sulfurreducens *pilB* deletion strains may be necessary.

**IMPORTANCE** Geobacter sulfurreducens is a model microbe for the study of biogeochemically and technologically significant processes, such as the reduction of Fe(III) oxides in soils and sediments, bioelectrochemical applications that produce electric current from waste organic matter or drive useful processes with the consumption of renewable electricity, direct interspecies electron transfer in anaerobic digestors and methanogenic soils and sediments, and metal corrosion. Elucidating the phenotypes associated with gene deletions is an important strategy for determining the mechanisms for extracellular electron transfer in G. sulfurreducens. The results reported here demonstrate that we cannot replicate the key phenotype reported for a gene deletion that has been central to the development of models for long-range electron transport in G. sulfurreducens.

## INTRODUCTION

Strains of Geobacter sulfurreducens and closely related *Geobacter* species produce some of the highest recorded current densities in microbial fuel cells, and *Geobacter* species are often the most abundant microorganisms within anode biofilms harvesting current in open systems, such as sediments or wastewater (1, 2). G. sulfurreducens has also served as a convenient model microbe for other extracellular electron transfer processes, such as Fe(III) oxide reduction, direct interspecies electron transfer (DIET), and corrosion (3–5). However, developing definitive models for extracellular electron transfer in G. sulfurreducens has been challenging. G. sulfurreducens produces a wide diversity of outer-surface redox-active proteins and electrically conductive filaments and can rapidly adapt expression of those outer-surface components in response to selective pressure that favors specific types of extracellular electron transfer or to mutations that disable electron transfer components (4, 6–9). Gene deletions designed to elucidate function may have an unintended negative impact on the expression of nontarget proteins (1).

There has been considerable debate over the role of electrically conductive nanofilaments emanating from G. sulfurreducens in long-range extracellular electron transport. In one model, filaments comprised of the multi-heme *c*-type cytochromes are the conduits for long-range electron transport (10, 11). Initially, it was proposed that filaments comprised of the multi-heme *c*-type cytochrome OmcS were responsible for long-range electron transport to electron-accepting electrodes (10). However, this hypothesis ignored the fact that deletion of *omcS* does not inhibit current production (12) and actually increases biofilm conductivity (13). Later, it was suggested that OmcZ, another multi-heme *c*-type cytochrome, was the conduit for long-range electron transport in current-producing biofilms (11). However, this hypothesis is not consistent with the observation that OmcZ is specially localized near the electrode surface (14) and thus not in a position to promote electron transport through the bulk of the biofilm. Furthermore, there is no correlation between OmcZ abundance and biofilm conductivity (13). For these and other reasons (4, 15, 16) cytochrome-based filaments do not appear to be the primary conduits for long-range extracellular electron transport in G. sulfurreducens.

An alternative hypothesis is that electrically conductive pili (e-pili) provide a route for long-range electron transport (4, 15, 16). Multiple lines of evidence demonstrate that PilA, the G. sulfurreducens pilin monomer, assembles into conductive filaments. Heterologous expression of the G. sulfurreducens PilA monomer in Pseudomonas aeruginosa (17) and Escherichia coli (18) yields e-pili with the same morphology and conductance as the e-pili recovered from G. sulfurreducens. G. sulfurreducens strains expressing PilA with peptide tags display filaments with those tags (19). Decreasing the aromatic amino acid content of the pilin expressed in G. sulfurreducens decreases pili conductivity, and increasing the abundance of aromatic amino acids increases conductivity (20–25). PilA monomers are recovered in filament preparations from G. sulfurreducens but only after harsh denaturation conditions, indicating that PilA is assembled into a highly stable filament (26).

Networks of electrically conductive pili were proposed to be the primary conduits for long-range electron transport to Fe(III) oxides (27), through conductive current-producing biofilms (28), and for DIET (7). One of the primary lines of evidence in these initial studies was the inhibition of long-range electron transport when the gene for PilA was deleted. However, it was subsequently found that deletion of *pilA* could negatively impact the localization of outer-surface multi-heme *c*-type cytochromes (29), which also play an important role in extracellular electron exchange (1, 3, 4). Furthermore, G. sulfurreducens pili may have additional functions, such as aiding in attachment to surfaces and biofilm formation (30). These concerns were eliminated with the development of G. sulfurreducens strains in which *pilA* was replaced with pilin genes designed to yield poorly conductive pili (20–23, 25). Such strains properly express outer-surface cytochromes (20, 21, 25) but are defective in Fe(III) oxide reduction and DIET and produce low current densities (20, 21, 23, 31), which are results consistent with e-pili serving as the primary conduit for long-range electron transport (4, 16).

An alternative strategy proposed to avoid concerns of proper outer-surface cytochrome expression is to delete the gene encoding PilB, the putative pilus assembly motor protein (23). However, the proposed lack of e-pili expression was only inferred from the expected impact of a *pilB* deletion on pili expression and not directly demonstrated (23). The PilB-deficient strain produced a maximum current (0.52 mA) that was half that of the wild-type strain (1.06 mA) (23). This is a substantially higher proportion of wild-type current production than achieved with a strain expressing poorly conductive pili, which generated only maximum currents that were ca. 15% of wild-type (20). The current production phenotype of the *pilB* deletion was evaluated in “batch mode,” in which the supply of the electron donor is provided only at the beginning of the incubation and is depleted over time. It is well known that maximum current production in G. sulfurreducens is best evaluated when cells are grown in anode chambers in which fresh medium is continuously supplied once current production begins (28). This method avoids the possibility of current production being inhibited prematurely due to a lack of electron donor or other medium constituents or an accumulation of toxic by-products over time (12).

**FIG 1:**
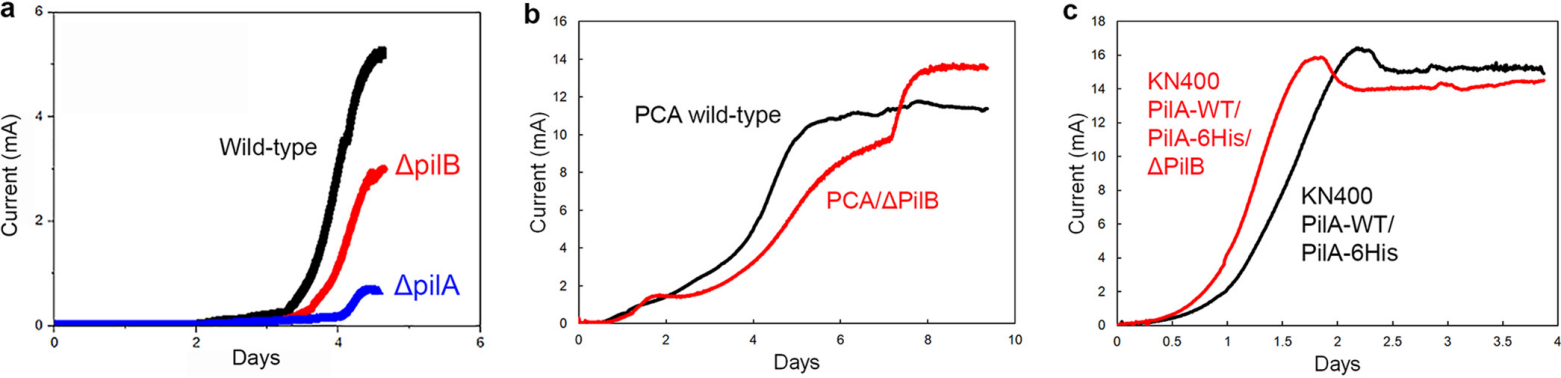
Current production of G. sulfurreducens strains. (a) Data image from reference 32 modified to label each of the different colored curves. (b) Current production for wild-type G. sulfurreducens strain PCA and G. sulfurreducens strain PCA/ΔPilB. The curve for the PCA/ΔPilB strain is representative of 9 replicates which all yielded a maximum current of ≥12 mA. (c) Current production for G. sulfurreducens KN400 strain PilA-WT/PilA-6×His and KN400 strain PilA-WT/PilA-6×His/ΔPilB. The curve for the PilA-WT/PilA-6×His/ΔPilB strain is representative of triplicates which all yielded a maximum current of ≥14 mA. Image in a is reproduced with permission.

A subsequent study further investigated a G. sulfurreducens strain in which the same *pilB* gene was deleted (32). As in the previous study, there was no evidence that this strain failed to produce pili. The *pilB*-deficient strain had an initial lag period that was ca. 10% longer than wild-type (ca. 88 h versus 80 h) prior to the initiation of rapid current increase (Fig. 1a). However, lag times are inherently variable even within replicates of the wild-type strain, and slight differences in the initial start of current production are not meaningful phenotypes for interpreting long-range electron transport mechanisms, which are required only after thick biofilms begin to form (33). Unfortunately, the incubation reported (32) was not long enough to determine whether the PilB-deficient strain produced the same maximum current as the wild-type strain. These results suggested that further evaluation of the impact of deleting *pilB* was warranted.

## RESULTS AND DISCUSSION

### High current densities in *pilB* deletion strains.

In order to further evaluate the impact of deleting *pilB*, the gene studied previously (23, 32) was deleted from the type strain of G. sulfurreducens (strain PCA), which is the same strain in which *pilB* was deleted in previous studies (23, 32).This strain was designated G. sulfurreducens strain PCA/ΔPilB. Current production was evaluated in anode chambers in which fresh medium was supplied continuously once current production began, as described previously (12). Under these optimized conditions, strain PCA/ΔPilB produced currents comparable to those of the wild-type (Fig. 1b).

The purpose of the deleting *pilB* in previous studies was to generate strains that did not express e-pili (23, 32). Therefore, we also deleted *pilB* in a strain designed to simplify e-pili visualization. G. sulfurreducens strain KN400 was selected for its superior growth on negatively poised anodes (34). It produces higher current densities (34) than strain PCA and biofilms with higher conductivity (13, 35), while expressing a lower abundance of outer-surface *c*-type cytochromes (34). A genetically modified strain of strain KN400, known as strain KN400 PilA-WT/PilA-6×His, expresses a wild-type PilA pilin monomer and a PilA monomer modified with a “His tag” (six histidines) at the carboxyl end (19). These pilin monomers assemble into e-pili emanating from cells that are identified with an immunogold/transmission electron microscopy (TEM) procedure that employs an antibody that recognizes the His tag (19). The e-pili with the His tag have conductivities comparable to those of wild-type e-pili, and the strain expressing the His tag e-pili produces current densities comparable to those of the wild-type KN400 strain (19). Deletion of *pilB* in strain KN400 PilA-WT/PilA-6×His yielded strain KN400/PilA-WT/PilA-6×His/ΔPilB. This strain produced current as well as the parental strain did (Fig. 1c).

Thus, our data do not support the claims from previous studies (23, 32) that deleting *pilB* negatively impacts G. sulfurreducens current production. This is an important consideration because diminished current production following deletion of *pilB* has served as the key phenotype supporting the claim that deletion of *pilB* is a strategy to evaluate the role of G. sulfurreducens e-pili in long-range electron transport for current production or DIET (23, 32).

### e-Pili expression in *pilB* deletion strains seems likely.

Previous studies have suggested that G. sulfurreducens can generate high current densities only when expressing e-pili (20, 21, 23, 36). Whenever genes that encode pilins expected to yield poorly conductive pili are expressed in G. sulfurreducens, current production and biofilm conductivity are reduced substantially even though outer-surface cytochromes are localized properly. Therefore, one possibility is that deletion of *pilB* does not prevent e-pili expression. A preliminary evaluation of this possibility suggests that e-pili expression is likely.

**FIG 2:**
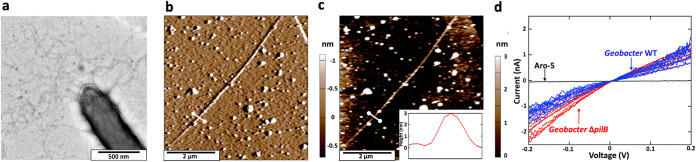
Expression of 3-nm-diameter electrically conductive filaments in the strain PCA/ΔPilB. (a) Transmission electron micrograph of a negatively stained cell. (b) Atomic force microscopy, noncontact topographical imaging with deflection output of a single filament, harvested from cells, laying on highly oriented pyrolytic graphite. (c) Height of the filament at the white line cross-section shown on the filament with the height profile shown in the inset. (d) Point mode IV spectroscopy of the individual G. sulfurreducens strain PCA/ΔPilB filaments shown in red overlaid with wild-type G. sulfurreducens in blue and G. sulfurreducens strain Aro-5 (in which the native PilA gene is replaced with a synthetic pilin gene designed to yield poorly conductive pili) in black. Data for pili from wild-type and strain Aro-5 are from reference 44.

Transmission electron micrographs of strain PCA/ΔPilB grown with fumarate as the electron acceptor revealed multiple filaments emanating from cells (Fig. 2a). Under these growth conditions, wild-type G. sulfurreducens expresses both e-pili and filaments comprised of OmcS, which have diameters of 3 nm and 4 nm, respectively (25). In studies with atomic force microscopy, only 10% of the filaments emanating from wild-type cells were found to be OmcS filaments (25), but it is difficult to unequivocally determine filament composition with transmission electron microscopy.

However, with atomic force microscopy, the distinction between 3-nm-diameter e-pili and 4-nm-diameter OmcS filaments is readily discerned (25). In addition to their characteristic 4-nm diameter, the OmcS filaments exhibit an axial periodicity of 20 nm that the 3-nm-diameter filaments lack. When a synthetic pilin designed to yield poorly conductive pili was expressed, there was no impact on the abundance or conductance of OmcS filaments, but the conductance of the 3-nm filaments was more than 100-fold lower (25). This result indicates that the 3-nm-diameter filaments are comprised of pilin. This conclusion was further supported by the finding that deleting the gene for OmcS and other outer-surface cytochromes yielded a strain that expressed only 3-nm-diameter filaments with a conductance similar to that observed in wild-type cells (25).

Therefore, filaments harvested from strain PCA/ΔPilB were examined with conducting probe atomic force microscopy, as described previously (37). Single filaments deposited on highly oriented pyrolytic graphite, which were identified in noncontact mode (Fig. 2b), had a height (Fig. 2c) of 3.0 ± 0.09 nm (mean ± standard deviation; *n* = 18; 6 individual points on 3 individual pili), consistent with the diameter of pili expressed in wild-type G. sulfurreducens (25) and the pili produced when the G. sulfurreducens PilA pilin gene is expressed heterologously in Pseudomonas aeruginosa (17) or Escherichia coli (18). The response of individual filaments in point mode current voltage (IV) spectroscopy was similar to that described previously for the e-pili of wild-type G. sulfurreducens (Fig. 2d) and the e-pili produced when the G. sulfurreducens PilA is expressed heterologously in E. coli (18). These results suggest that e-pili continued to be expressed after *pilB* was deleted.

**FIG 3:**
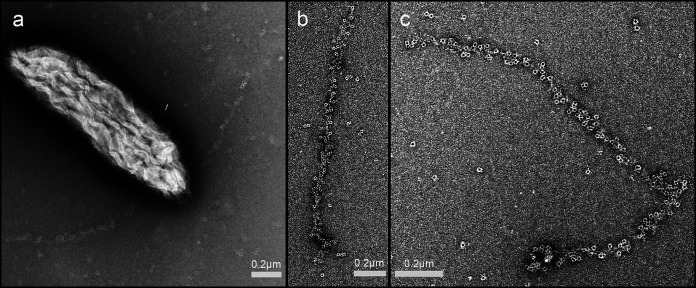
Immunogold labeling of pilin-containing filaments from strain PilA-WT/PilA-6×His/ΔPilB. (a) Labeled filaments emanating from cell. (b and c) Higher magnification of labeled filaments.

In order to further evaluate the possibility of e-pili expression following deletion of *pilB*, the G. sulfurreducens KN400 gene encoding the same protein identified previously as PilB (23) was deleted from the KN400 strain PilA-WT/PilA-6×His to yield strain KN400/PilA-WT/PilA-6×His/ΔPilB. His tag immunogold labeling of strain PilA-WT/PilA-6×His/ΔPilB revealed labeled filaments (Fig. 3) similar to those previously reported (19) for the strain without the *pilB* deletion. As noted previously for the strain without the *pilB* deletion (19), no unlabeled filaments were observed, suggesting that e-pili were the primary filaments expressed. These results further suggest that e-pili comprised of PilA monomers continued to be expressed when *pilB* was deleted.

### Possibilities for expression of e-pili in strain ΔPilB.

G. sulfurreducens PilB is homologous to other bacterial PilB ATPases and contains highly conserved features, such as Walker A and B motifs, an Asp box, a His box, Arg fingers, and a tetracysteine Zn^2+^-binding motif (38). The gene (GSU1491) for PilB is located in a cluster, including *pilT-4*, *pilC*, *pilS*, *pilR*, and *pilA* on the G. sulfurreducens genome (27). However, there are multiple genes within the G. sulfurreducens genome that might function as a PilB ATPase or compensate for deletion of the gene for the PilB ATPase, which were previously reported (39) to be highly expressed in current-producing biofilms (Table 1). Another possibility is that type IV e-pili could be assembled by a type II protein secretion system, which is similar to the type IV pilus assembly system in composition and structure and polymerizes pseudopilins (40, 41).

**TABLE 1:** PilB homologs in G. sulfurreducens

Gene	Description^*a*^	Identity (%)^*b*^	Transcript abundance^*c*^
GSU1491	Type IV pilus biogenesis ATPase PilB	100	9.91
GSU0328	Type II secretion system ATPase GspE	45	10.12
GSU0435	PilB/PulE/GspE family ATPase	26	6.86
GSU1783	Type II secretion system ATPase PulE	38	9.70
GSU2609	PilB/PulE/GspE family ATPase	40	9.50

a Description is from NCBI reference sequence of G. sulfurreducens.

b Identity is based on NCBI BLAST (https://blast.ncbi.nlm.nih.gov/Blast.cgi) with PilB (GSU1491) as the query.

c The values (log_2_ signal) of transcript abundance are from those for growth on a current-producing anode (39).

### Implications.

The results presented here demonstrate that deleting *pilB* does not prevent the generation of high current densities by G. sulfurreducens. This result contrasts with previous studies that suggested that deleting *pilB* diminishes current production (23, 32). Diminished current production was assumed to result from the *pilB* deletion inhibiting expression of e-pili (23, 32). The assumed lack of e-pili expression was used to interpret the role of G. sulfurreducens e-pili in not only current production but also DIET (23, 32). Our results suggest that before the phenotypes of *pilB* deletion mutants are interpreted as a response to a lack of e-pili, the assumed lack of e-pili will need to be thoroughly documented. Our findings that strain PCA/ΔPilB expressed filaments with a morphology and conductance similar to those reported for e-pili and that strain PilA-WT/PilA-6×His/ΔPilB expressed filaments comprised of His tag pilin monomers suggest that e-pili continue to be expressed even when *pilB* is deleted.

One of the primary benefits attributed to deleting *pilB* in G. sulfurreducens was that it yielded a strain without e-pili in which the outer-surface *c*-type cytochromes were localized properly. The same result is achieved with the expression of pilin monomers that yield poorly conductive pili (20, 21, 25) with the added benefit that other possible functions of e-pili, such as aiding in attachment to surfaces and biofilm formation (30), are retained.

## MATERIALS AND METHODS

### Strains and growth conditions.

G. sulfurreducens strains were grown under anaerobic conditions at 30°C in a defined medium with acetate as the electron donor and fumarate as the electron acceptor as described previously (42) unless otherwise mentioned. Escherichia coli NEB 10-beta (New England BioLabs) was used for plasmid preparation and grown in medium supplemented as instructed by the manufacturer, with appropriate antibiotics if necessary.

### Construction of ΔPilB strains.

To construct G. sulfurreducens strain PCA/ΔPilB, the *pilB* gene (GSU1491) was replaced by a kanamycin-resistance gene in G. sulfurreducens PCA via the double-crossover homologous recombination as described previously (42). Flanking DNA fragments were amplified by PCR with primer pairs ATCTCTAGATTCCTCATAAATCGGCCATC (XbaI site is underlined)/TCTGAATTCAGTCTGCTAGCCTGCATAG (EcoRI site is underlined) for the upstream region and TCTAAGCTTCTATCAGGTAATGCCCATG (HindIII site is underlined)/TCTGGTACCTCGATGGTCACAATATGATC (KpnI site is underlined) for the downstream region. The G. sulfurreducens PCA chromosome DNA was used as the template. The DNA fragment of the kanamycin-resistance gene was amplified by PCR as described previously (43). These PCR products were digested with restriction enzymes, ligated, and cloned in a plasmid. The plasmid constructed was linearized by XbaI. The linearized DNA fragment was used for electroporation. The deletion of the *pilB* gene and replacement with the kanamycin resistance gene were verified by PCR with primer pairs TTCCTCATAAATCGGCCATC/GCAGTCTTGATGGACGACTC and TTCCTCATAAATCGGCCATC/ACATTCATCCCAGGTGGCAC, respectively.

To construct strain KN400-PilA-WT/PilA-6×His/ΔPilB, the *pilB* gene (KN400_1518) was replaced by the kanamycin resistance gene in KN400-PilA-WT/PilA-6×His as described above, except that the G. sulfurreducens KN400 chromosome DNA was used as the template for PCR. There are 3 nucleotide differences between PCA and KN400 in the downstream region used for the recombination.

### Current production.

Current production was determined in previously described bioelectrochemical systems (12) with acetate as the electron donor and positively poised (300 mV versus Ag/AgCl) graphite anodes as the electron acceptor. Once current production was initiated, the anode chamber received a steady input of fresh medium as described previously (12).

### Atomic force microscopy.

Atomic force microscopy (AFM) was carried out, as described previously, with minor modifications (37). Briefly, 100 μl of a pili preparation sheared from cells was drop cast onto highly oriented pyrolytic graphite (HOPG) and allowed to set for 10 min, after which it was washed twice with 100 μl of deionized water and blotted dry. The sample was equilibrated with atmospheric humidity for at least 2 h before subsequent measurements. AFM was performed on an Oxford Instruments Cypher ES environmental AFM instrument in ORCA electrical mode equipped with a platinum/iridium-coated Arrow-ContPT tip with a 0.2-N/m force constant (NanoWorld AG, Neuchâtel, Switzerland). Topographical identification of the pili was achieved in contact mode, with a set point of 0.002 V. Conductive measurements were acquired using a 0.002-V set point (equating to an applied force of 2 nN), and IV curves of a ±0.6 V voltage sweep at a frequency of 0.99 Hz were generated for three spatially different points on three independent pili. To test if the AFM tip was connected correctly to the AFM and to ensure that it was clear of debris, the tip was touched periodically to the surface of the HOPG and an IV curve was generated. Additionally, to test for probe clearance from the sample/substrate, the tip was raised off the surface and an IV curve generated to ensure no signal.

### Transmission electron microscopy and immunogold labeling.

Immunogold labeling was conducted with the 6× His Tag polyclonal antibody as the primary antibody and the anti-rabbit IgG gold (10 nm) antibody as the secondary antibody, as described previously (19). For transmission electron microscopy, 7 μl of the sample was drop cast on a plasma-sterilized, 400-mesh copper carbon-coated ultralight grid for 10 minutes. Excess liquid was wicked off and the grid was stained with 3 μl of 2% uranyl acetate for 15 to 20 seconds before excess liquid was removed and air dried. Grids were examined with transmission electron microscopy on a FEI Tecnai 12 instrument at 120 kV or a JEOL 2000fx instrument at 200 kV.

### Data availability.

The G. sulfurreducens genome sequence is available at the NCBI database (https://www.ncbi.nlm.nih.gov/) under reference sequence NC_002939.5.

## References

[1] Lovley DR, Ueki T, Zhang T, Malvankar NS, Shrestha PM, Flanagan K, Aklujkar M, Butler JE, Giloteaux L, Rotaru A-E, Holmes DE, Franks AE, Orellana R, Risso C, Nevin KP. 2011. Geobacter: the microbe electric’s physiology, ecology, and practical applications. Adv Microb Physiol 59:1–100. doi:10.1016/B978-0-12-387661-4.00004-5. 22114840

[2] Logan BE, Rossi R, Ragab A, Saikaly PE. 2019. Electroactive microorganisms in bioelectrochemical systems. Nat Rev Microbiol 17:307–319. doi:10.1038/s41579-019-0173-x. 30846876

[3] Shi L, Dong H, Reguera G, Beyenal H, Lu A, Liu J, Yu H-Q, Fredrickson JK. 2016. Extracellular electron transfer mechanisms between microorganisms and minerals. Nat Rev Microbiol 14:651–662. doi:10.1038/nrmicro.2016.93. 27573579

[4] Lovley DR, Holmes DE. 2021. Electromicrobiology: the ecophysiology of phylogenetically diverse electroactive microorganisms. Nat Rev Microbiol doi:10.1038/s41579-021-00597-6. 34316046

[5] Lekbach Y, Liuc T, Li Y, Moradia M, Doue W, Xu D, Smith JA, Lovley DR. 2021. Microbial corrosion of metals-the corrosion microbiome. Adv Microb Physiol 78:317–390. doi:10.1016/bs.ampbs.2021.01.002. 34147188

[6] Leang C, Adams LA, Chin K-J, Nevin KP, Methé BA, Webster J, Sharma ML, Lovley DR. 2005. Adaption to disruption of electron tranfer pathway for Fe(III) reduction in Geobacter sulfurreducens. J Bacteriol 187:5918–5926. doi:10.1128/JB.187.17.5918-5926.2005. 16109933 PMC1196151

[7] Summers ZM, Fogarty H, Leang C, Franks AE, Malvankar NS, Lovley DR. 2010. Direct exchange of electrons within aggregates of an evolved syntrophic co-culture of anaerobic bacteria. Science 330:1413–1415. doi:10.1126/science.1196526. 21127257

[8] Tremblay P-L, Summers ZM, Glaven RH, Nevin KP, Zengler K, Barrett CL, Qiu Y, Palsson BO, Lovley DR. 2011. A c-type cytochrome and a transcriptional regulator responsible for enhanced extracellular electron transfer in *Geobacter sulfurreducens* uncovered by adapative evolution. Environ Microbiol 13:13–23. doi:10.1111/j.1462-2920.2010.02302.x. 20636372

[9] Smith JA, Tremblay P-L, Shrestha PM, Snoeyenbos-West OL, Franks AE, Nevin KP, Lovley DR. 2014. Going wireless: Fe(III) oxide reduction without pili by Geobacter sulfurreducens strain JS-1. Appl Environ Microbiol 80:4331–4340. doi:10.1128/AEM.01122-14. 24814783 PMC4068678

[10] Wang F, Gu Y, O'Brien JP, Yi SM, Yalcin SE, Srikanth V, Shen C, Vu D, Ing NL, Hochbaum AI, Egelman EH, Malvankar NS. 2019. Structure of microbial nanowires reveals stacked hemes that transport electrons over micrometers. Cell 177:361–369. doi:10.1016/j.cell.2019.03.029. 30951668 PMC6720112

[11] Yalcin SE, O’Brien JP, Gu Y, Reiss K, Yi SM, Jain R, Srikanth V, Dahl DJ, Huynh W, Vu D, Acharya A, Chaudhuri S, Varga T, Batista VS, Malvankar NS. 2020. Electric field stimulates production of highly conductive microbial OmcZ nanowires. Nat Chem Biol 16:1136–1142. doi:10.1038/s41589-020-0623-9. 32807967 PMC7502555

[12] Nevin KP, Kim B-C, Glaven RH, Johnson JP, Woodard TL, Methé BA, DiDonato RJ, Jr, Covalla SF, Franks AE, Liu A, Lovley DR. 2009. Anode biofilm transcriptomics reveals outer surface components essential for high current power production in Geobacter sulfurreducens fuel cells. PLoS One 4:e5628. doi:10.1371/journal.pone.0005628. 19461962 PMC2680965

[13] Malvankar NS, Tuominen MT, Lovley DR. 2012. Lack of involvement of c-type cytochromes in long-range electron transport in microbial biofilms and nanowires of *Geobacter sulfurreducens*. Energy Environ Sci 5:8651–8659. doi:10.1039/c2ee22330a.

[14] Inoue K, Leang C, Franks AE, Woodard TL, Nevin KP, Lovley DR. 2011. Specific localization of the *c*-type cytochrome OmcZ at the anode surface in current-producing biofilms of *Geobacter sulfurreducens*. Environ Microbiol Rep 3:211–217. doi:10.1111/j.1758-2229.2010.00210.x. 23761253

[15] Lovley DR, Walker DJF. 2019. Geobacter protein nanowires. Front Microbiol 10:2078. doi:10.3389/fmicb.2019.02078. 31608018 PMC6771412

[16] Lovley DR, Holmes DE. 2020. Protein Nanowires: the electrification of the microbial world and maybe our own. J Bacteriol 202:e00331-20. doi:10.1128/JB.00331-20. PMC7515249 32747429

[17] Liu X, Wang S, Xu A, Zhang L, Liu H, Ma LZ. 2019. Biological synthesis of high-conductive pili in aerobic bacterium Pseudomonas aeruginosa. Appl Microbiol Biotechnol 103:1535–1544. doi:10.1007/s00253-018-9484-5. 30523372

[18] Ueki T, Walker DJF, Woodard TL, Nevin KP, Nonnenmann S, Lovley DR. 2020. An *Escherichia coli* chassis for production of electrically conductive protein nanowires. ACS Synth Biol 9:647–654. doi:10.1021/acssynbio.9b00506. 32125829

[19] Ueki T, Walker DJF, Tremblay P-L, Nevin KP, Ward JE, Woodard TL, Nonnenmann SS, Lovley DR. 2019. Decorating the outer surface of microbially produced protein nanowires with peptides. ACS Synth Biol 8:1809–1817. doi:10.1021/acssynbio.9b00131. 31298834

[20] Vargas M, Malvankar NS, Tremblay P-L, Leang C, Smith JA, Patel P, Snoeyenbos-West O, Synoeyenbos-West O, Nevin KP, Lovley DR. 2013. Aromatic amino acids required for pili conductivity and long-range extracellular electron transport in Geobacter sulfurreducens. mBio 4:e00105-13. doi:10.1128/mBio.00105-13. 23481602 PMC3604773

[21] Liu X, Tremblay P-L, Malvankar NS, Nevin KP, Lovley DR, Vargas M. 2014. A Geobacter sulfurreducens strain expressing Pseudomonas aeruginosa type IV pili localizes OmcS on pili but Is deficient in Fe(III) oxide reduction and current production. Appl Environ Microbiol 80:1219–1224. doi:10.1128/AEM.02938-13. 24296506 PMC3911229

[22] Adhikari RY, Malvankar NS, Tuominen MT, Lovley DR. 2016. Conductivity of individual Geobacter pili. RSC Adv 6:8354–8357. doi:10.1039/C5RA28092C.

[23] Steidl RJ, Lampa-Pastirk S, Reguera G. 2016. Mechanistic stratification in electroactive biofilms of Geobacter sulfurreducens mediated by pilus nanowires. Nat Commun 7:12217. doi:10.1038/ncomms12217. 27481214 PMC4974642

[24] Tan Y, Adhikari RY, Malvankar NS, Ward JE, Woodard TL, Nevin KP, Lovley DR. 2017. Expressing the Geobacter metallireducens PilA in Geobacter sulfurreducens yields pili with exceptional conductivity. mBio 8:e02203-16. doi:10.1128/mBio.02203-16. 28096491 PMC5241403

[25] Liu X, Walker DJF, Nonnenmann S, Sun D, Lovley DR. 2021. Direct observation of electrically conductive pili emanating from Geobacter sulfurreducens. mBio 12:e02209-21. doi:10.1128/mBio.02209-21. 34465020 PMC8406130

[26] Tan Y, Adhikari RY, Malvankar NS, Ward JE, Nevin KP, Woodard TL, Smith JA, Snoeyenbos-West OL, Franks AE, Tuominen MT, Lovley DR. 2016. The low conductivity of Geobacter uraniireducens pili suggests a diversity of extracellular electron transfer mechanisms in the genus Geobacter. Front Microbiol 7:980. doi:10.3389/fmicb.2016.00980. 27446021 PMC4923279

[27] Reguera G, McCarthy KD, Mehta T, Nicoll JS, Tuominen MT, Lovley DR. 2005. Extracellular electron transfer via microbial nanowires. Nature 435:1098–1101. doi:10.1038/nature03661. 15973408

[28] Reguera G, Nevin KP, Nicoll JS, Covalla SF, Woodard TL, Lovley DR. 2006. Biofilm and nanowire production leads to increased current in *Geobacter sulfurreducens* fuel cells. Appl Environ Microbiol 72:7345–7348. doi:10.1128/AEM.01444-06. 16936064 PMC1636155

[29] Izallalen M, Glaven RH, Mester T, Nevin KP, Franks AE, Lovley DR. 2008. Going wireless? Additional phenotypes of a pilin-deficient mutant weaken the genetic evidence for the role of microbial nanowires in extracellular electron transfer. 108th Annual Meeting of the American Society for Microbiology, Boston, MA.

[30] Reguera G, Pollina RB, Nicoll JS, Lovley DR. 2007. Possible non-conductive role of *Geobacter sulfurreducens* pili nanowires in biofilm formation. J Bacteriol 189:2125–2127. doi:10.1128/JB.01284-06. 17158673 PMC1855775

[31] Ueki T, Nevin KP, Rotaru A-E, Wang L-Y, Ward JE, Woodard TL, Lovley DR. 2018. Geobacter strains expressing poorly conductive pili reveal constraints on direct interspecies electron transfer mechanisms. mBio 9:e01273-18. doi:10.1128/mBio.01273-18. 29991583 PMC6050967

[32] Liu X, Zhuo S, Rensing C, Zhou J. 2018. Syntrophic growth with direct interspecies electron transfer between pili-free Geobacter species. ISME J 12:2142–2151. doi:10.1038/s41396-018-0193-y. 29875437 PMC6092431

[33] Lovley DR. 2017. Electrically conductive pili: biological function and potential applications in electronics. Curr Opin Electrochem 4:190–198. doi:10.1016/j.coelec.2017.08.015.

[34] Yi H, Nevin KP, Kim B-C, Franks AE, Klimes A, Tender LM, Lovley DR. 2009. Selection of a variant of *Geobacter sulfurreducens* with enhanced capacity for current production in microbial fuel cells. Biosens Bioelectron 24:3498–3503. doi:10.1016/j.bios.2009.05.004. 19487117

[35] Malvankar NS, Vargas M, Nevin KP, Franks AE, Leang C, Kim B-C, Inoue K, Mester T, Covalla SF, Johnson JP, Rotello VM, Tuominen MT, Lovley DR. 2011. Tunable metallic-like conductivity in nanostructured biofilms comprised of microbial nanowires. Nat Nanotechnol 6:573–579. doi:10.1038/nnano.2011.119. 21822253

[36] Walker DJF, Adhikari RY, Holmes DE, Ward JE, Woodard TL, Nevin KP, Lovley DR. 2018. Electrically conductive pili from genes of phylogenetically diverse microorganisms. ISME J 12:48–58. doi:10.1038/ismej.2017.141. 28872631 PMC5739001

[37] Walker DJF, Nevin KP, Holmes DE, Rotaru A-E, Ward JE, Woodard TL, Zhu J, Ueki T, Nonnenmann SS, McInerney MJ, Lovley DR. 2020. Syntrophus conductive pili demonstrate that common hydrogen-donating syntrophs can have a direct electron transfer option. ISME J 14:837–846. doi:10.1038/s41396-019-0575-9. 31896792 PMC7031330

[38] Solank V, Kapoor S, Thakur KG. 2018. Structural insights into the mechanism of Type IVa pilus extension and retraction ATPase motors. FEBS J 285:3402–3421. doi:10.1111/febs.14619. 30066435

[39] Qiu Y, Cho BK, Park YS, Lovley D, Palsson BO, Zengler K. 2010. Structural and operational complexity of the Geobacter sulfurreducens genome. Genome Res 20:1304–1311. doi:10.1101/gr.107540.110. 20592237 PMC2928509

[40] Cisneros DA, Pehau-Arnaudet GOF. 2012. Heterologous assembly of type IV pili by a type II secretion system reveals the role of minor pilins in assembly initiation. Mol Microbiol 86:805–818. doi:10.1111/mmi.12033. 23006128

[41] Sauvonnet N, Vignon G, Pugsley AP, Gounon P. 2000. Pilus formation and protein secretion by the same machinery in *Escherichia coli*. EMBO J 19:2221–2228. doi:10.1093/emboj/19.10.2221. 10811613 PMC384360

[42] Coppi MV, Leang C, Sandler SJ, Lovley DR. 2001. Development of a genetic system for *Geobacter sulfurreducens*. Appl Environ Microbiol 67:3180–3187. doi:10.1128/AEM.67.7.3180-3187.2001. 11425739 PMC92998

[43] Ueki T, Lovley DR. 2010. Genome-wide gene regulation in of biosynthesis and energy generation by a novel transcriptional repressor in Geobacter species. Nucleic Acids Res 38:810–821. doi:10.1093/nar/gkp1085. 19939938 PMC2817479

[44] Walker DJF, Martz E, Holmes DE, Zhou Z, Nonnenmann SS, Lovley DR. 2019. The archaellum of Methanospirillum hungatei is electrically conductive. mBio 10:e00579-19. doi:10.1128/mBio.00579-19. 30992355 PMC6469973

